# Corneal Nerve Abnormalities in Painful Dry Eye Disease Patients

**DOI:** 10.3390/biomedicines9101424

**Published:** 2021-10-09

**Authors:** Adrian Guerrero-Moreno, Hong Liang, Nathan Moreau, Jade Luzu, Ghislaine Rabut, Stéphane Melik Parsadaniantz, Antoine Labbé, Christophe Baudouin, Annabelle Réaux-Le Goazigo

**Affiliations:** 1 Institut de la Vision, INSERM, CNRS, Sorbonne Université, 17 rue Moreau, 75012 Paris, France; adrian.guerrero@inserm.fr (A.G.-M.); hliang-bouttaz@15-20.fr (H.L.); nthmoreau@gmail.com (N.M.); stephane.melik-parsadaniantz@inserm.fr (S.M.P.); cbaudouin@15-20.fr (C.B.); 2 CHNO des Quinze-Vingts, INSERM-DGOS CIC 1423, 17 rue Moreau, 75012 Paris, France; jluzu@15-20.fr (J.L.); grabut@15-20.fr (G.R.); alabbe@15-20.fr (A.L.); 3 Department of Ophthalmology, Ambroise Paré Hospital, AP-HP, University of Versailles Saint-Quentin-en-Yvelines, 9 Avenue Charles de Gaulle, 92100 Boulogne-Billancourt, France

**Keywords:** neuropathic pain, nerve abnormalities, cornea, dry eye, microneuroma

## Abstract

**Background**: This study aimed to compare the corneal nerve structural abnormalities detected using in vivo confocal microscopy (IVCM) in patients with neuropathic corneal pain (NCP) secondary to primary meibomian gland dysfunction (MGD) or autoimmune dry eye (AIDE). **Methods**: A two-stage retrospective nested case–control study was conducted. First, data from patients with either MGD or AIDE were assessed, selecting only cases with no corneal pain (VAS = 0) or severe pain (VAS ≥ 8). Ocular signs and symptoms of the 238 selected patients were compared between painful and painless cases. Next, painful patients with no corneal damage (Oxford score ≤ 1) were selected within each study group, defining the cases with NCP (i.e., “pain without stain”). IVCM images from all groups were compared with prospectively-recruited healthy controls, focusing on dendritiform cell density and nerve abnormalities (density, tortuosity, microneuromas). **Results**: AIDE patients had more ocular signs/symptoms than MGD patients. Compared with healthy controls, AIDE-related NCP patients showed increased nerve tortuosity and number of neuromas, whereas MGD-related NCP patients had reduced nerve density and increased number, perimeter, and area of microneuromas. Microneuromas were also observed in healthy controls. Furthermore, a higher number of microneuromas was found in MGD-related NCP compared to AIDE-related NCP or painless MGD. **Conclusions**: MGD-related NCP was associated with significantly more corneal nerve abnormalities than AIDE-related NCP or healthy controls. Although IVCM can be useful to detect NCP-related corneal nerve changes in such patients, the diagnosis of dry eye disease-related NCP will require an association of several IVCM-based criteria without relying solely on the presence of microneuromas.

## 1. Introduction

Dry eye symptoms are among the most frequent complaints observed in general ophthalmological practice. They include visual disturbances, discomfort, and pain. When such symptoms become chronic, they can alter the patient’s quality of life, especially considering the absence of efficient treatments to date [1,2,3,4]. 

Dry eye disease (DED) is “a multifactorial disease of the ocular surface characterized by a loss of homeostasis of the tear film, and accompanied by ocular symptoms, in which tear film instability and hyperosmolarity, ocular surface inflammation and damage, and neurosensory abnormalities play etiological roles”, as defined in 2017 by the TFOS Dry Eye Workshop [5]. DED can be subclassified as aqueous deficient dry eye (ADDE) and/or evaporative dry eye (EDE) based on the underlying predominant pathophysiological mechanism involved, each group including multiple etiologies and risk factors of DED. The most common etiologies of DED are autoimmune-associated DED (AIDE, e.g., Sjögren syndrome) in ADDE and meibomian gland dysfunction (MGD) in EDE [6]. 

As encompassed in this definition, recent studies have focused on the role of neurosensory abnormalities in the pathophysiology and semiology of DED [1,7,8,9]. Indeed, reduced tear secretion and/or tear film instability lead to inflammation and peripheral nerve damage, triggering sensitization and abnormal activity in corneal nerves, evoking painful symptoms of dry eye associated with dryness, itching, and foreign body sensation [1]. 

Clinical diagnosis of DED is based on the assessment of these symptoms, but more importantly, on the observation of signs such as ocular surface damage (corneal stain), tear volume, and tear film hyperosmolarity [10]. However, there is a discordance between symptoms and signs in an important proportion of patients. Among them, some patients can present asymptomatic ocular surface alterations, and conversely, they can be highly symptomatic without obvious signs of ocular surface damage (i.e.,“pain without stain”) [9,11,12]. In the second case, patients present with complaints of burning, stinging, eye-ache, photophobia, or severe eye pain without significant findings on slit-lamp examination [8,13]. The concept of “neuropathic corneal pain” (NCP), increasingly investigated in the past 20 years in clinical research [4,14], has been suggested to play a central role in those cases. NCP represents a current clinical challenge, as those patients are commonly not responding to classic dry eye treatments [15], and the ocular pathology is often associated with depressive disorders [16]. 

In the clinical setting, NCP has been investigated using ocular surface pain questionnaires (such as the Ocular Pain Assessment Survey [OPAS]) [17], corneal esthesiometry, proparacaine challenge test (to ascertain the peripheral and/or central origin of the NCP) [18], and finally, in vivo confocal microscopy (IVCM), allowing non-invasive imaging of the cornea at the cellular level [14,19,20,21,22,23].

IVCM has been used to image subbasal nerve attributes in various ocular surface diseases, including DED [24,25,26]. IVCM has identified subbasal nerve alterations such as reduced nerve density, increased tortuosity, reflectivity, and presence of microneuromas [27,28,29,30]. Although it has recently been suggested that the visualization of microneuromas in IVCM could represent an objective biomarker for the diagnosis of NCP [31], there are scarce data regarding the IVCM-assessed ultrastructural characteristics of corneal nerves and microneuromas in patients with DED-related NCP. In particular, no study has investigated the potential differences in structural corneal nerve characteristics within the various ADDE/EDE etiological subgroups. As with other neuropathic pain conditions, proper phenotyping within the respective patient clusters is a mandatory prerequisite for tailored pharmacological management [32]. 

This retrospective clinical study thus aimed to investigate the clinical profiles of painful DED patients and the corneal nerve ultrastructural abnormalities in two subsets of DED-related NCP patients (MGD and AIDE) using IVCM.

## 2. Materials and Methods

### 2.1. Study Design 

**Figure 1:**
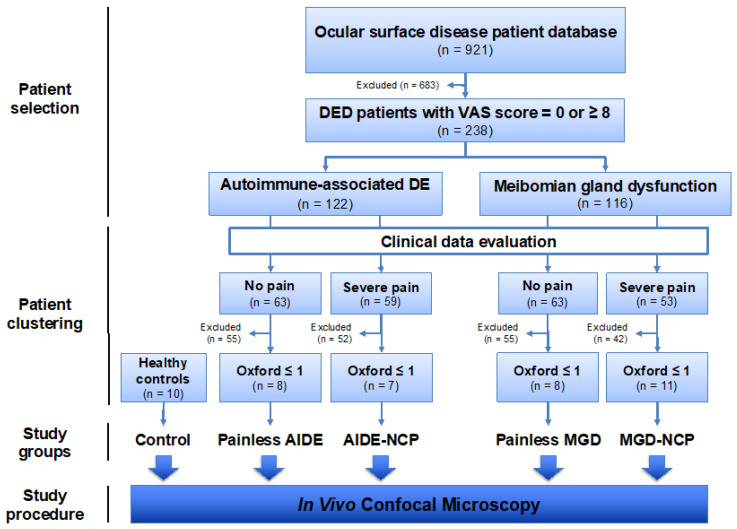
Retrospective nested case–control study flowchart.

To further elucidate the various corneal nerve changes associated with dry eye diseases, a retrospective nested case–control study was performed to assess the clinical features of patients suffering from primary MGD and AIDE and severe corneal pain, compared with their respective painless counterparts. Ultrastructural corneal characteristics were studied among patients presenting NCP characteristics (high pain score and absence of clinical signs) and compared with their painless counterparts and healthy volunteers. The study flowchart is summarized in Figure 1. 

### 2.2. Patient Cohorts

#### 2.2.1. Patient Selection for Clinical Features Study

This retrospective single-center study was conducted at the Center for Clinical Investigation (CIC INSERM 1423) of the Quinze-Vingts National Ophthalmology Hospital, Paris, France, between March 2012 and January 2020. The study was conducted in accordance with the tenets of the Declaration of Helsinki and approved by the Ethics Committee CPP-Ile de France (Number 10793). All patients gave written consent to use their data for research purposes.

This study included 921 patients with various ocular surface diseases. All patients had systematic pain history and ocular examination as part of their routine workup, and the clinical data were systematically collected in a secure spreadsheet (in adherence with national guidelines on data collection and protection). Patients were included in the study based on the following criteria: (1) a diagnosis of primary MGD or of an AIDE was made; (2) the patient reported no ocular pain (visual analog scale [VAS] score = 0) or severe ocular pain (VAS score ≥ 8) during history. 

#### 2.2.2. Patient Clustering for Ultrastructural IVCM Study of the Cornea

Previously selected patients were clustered into one of two study groups (MGD- or AIDE-related NCP) or two control groups (painless MGD or painless AIDE) based on the following diagnostic criteria. 

##### Dry-Eye Disease-Related Neuropathic Corneal Pain Diagnostic Criteria

In the absence of validated diagnostic criteria, NCP was diagnosed in patients with DED who presented severe pain in the absence or with minimal corneal damage, in adherence with the relevant literature [2,3,14,31]. Patients were thus diagnosed with DED-related NCP if they presented the following criteria: (1) a diagnosis of primary MGD or AIDE was made, accordingly to relevant diagnostic criteria; (2) the patient reported severe ocular pain (VAS score ≥ 8); (3) clinical examination revealed low corneal damage (Oxford score ≤ 1).

##### Painless Dry-Eye Disease Diagnostic Criteria

Patients with painless primary MGD or painless AIDE served as control conditions to their painful neuropathic counterparts (MGD-related NCP and AIDE-related NCP, respectively). Patients were thus diagnosed with painless DED if they presented the following criteria: (1) a diagnosis of primary MGD or AIDE was made, accordingly to relevant diagnostic criteria; (2) the patient reported no ocular pain (VAS score = 0); (3) clinical examination revealed low corneal damage (Oxford score ≤ 1).

##### Study Conditions for IVCM Study

Meibomian Gland Dysfunction-Related Neuropathic Corneal Pain

Of the previously included 116 MGD patients, 53 reported severe pain. Patients without corneal injury (Oxford score ≤ 1) and a minimum of 30 IVCM images from the subbasal plexus were selected for the IVCM study, excluding 42 of those 53 patients. The final MGD-NCP group thus comprised 11 patients (with severe pain and low corneal damage) (Figure 1).

Autoimmune-Associated Dry Eye-Related Neuropathic Corneal Pain

Of the previously included 122 AIDE patients, 59 reported severe pain. Patients without corneal damage (Oxford score ≤ 1) and a minimum of 30 IVCM images from the subbasal plexus were selected, excluding 52 of those 59 patients. The final AIDE-NCP group thus comprised 7 patients (Figure 1).

##### Study Controls 

Painless Meibomian Gland Dysfunction

Of the previously included 116 MGD patients, 63 reported no pain. To provide a comparable control condition to the neuropathic pain condition (MGD-related NCP), patients without corneal damage (Oxford score ≤ 1) and a minimum of 30 IVCM images from the subbasal plexus were selected (as in the relevant study group). The final painless MGD group thus comprised 8 patients (no pain and no corneal damage).

Painless Autoimmune-Associated Dry Eye

Of the previously included 122 AIDE patients, 63 reported no pain. To provide a comparable control condition to the neuropathic pain condition (AIDE-related NCP), patients without corneal damage (Oxford score ≤ 1) and a minimum of 30 IVCM images from the subbasal plexus were selected (as in the relevant study group). The final painless AIDE group thus comprised 8 patients (with no pain and no corneal damage).

Healthy Controls

Healthy volunteers (*n* = 10) were prospectively recruited and paired in age and sex with the various study groups to provide healthy controls for ultrastructural corneal analysis.

### 2.3. Clinical Data Evaluation

#### 2.3.1. General Data

Patient age, gender, and dry eye diagnosis were recorded systematically in all patients.

#### 2.3.2. Patient History

We investigated the VAS (scored from 0 to 10) for quantifying ocular pain score, the ocular surface disease index (OSDI, scored from 0 to 100), and frequency of self-reported adverse ocular surface symptoms such as itching, burning, foreign body sensation or dryness sensation (scored as never, rarely, sometimes, always).

#### 2.3.3. Ocular Examination

Dry eye signs were assessed with fluorescein corneal staining (Oxford score, 0 to 5), tear break-up time (BUT, seconds), non-invasive keratograph tear break-up time (NIKBUT, seconds), Shirmer test (millimeters of wetting after 5 min), tear meniscus length (millimeters), and tear osmolarity (mOsmol/L).

### 2.4. In Vivo Confocal Microscopy: Image Selection and Analysis

Corneal IVCM images were obtained using the Rostock Cornea Module (RCM) of the Heidelberg Retina Tomograph II (Heidelberg Engineering GmbH, Heidelberg, Germany) as previously described [28,29]. All images were obtained by experienced ophthalmologists, following the prior administration of one drop of topical anesthetic (oxybuprocaine 0.4%, MSD-Chibret, Paris, France). The x-y position and depth (z) of the optical section were controlled manually, with the focus position (in μm) automatically calculated by the HRT II/RCM, allowing the selection of high-quality representative images. Each IVCM-generated image comprised an area of 384 × 384 pixels (400 μm × 400 μm), focused on the central cornea.

#### Image Selection and Analysis

**Figure 2:**
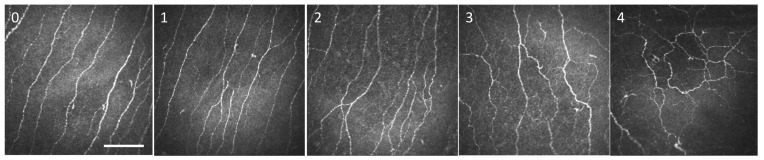
Corneal nerve fiber tortuosity scale graded from (**0**) to (**4**) based on in vivo confocal microscopy image. Scale bar = 100 μm.

**Figure 3:**
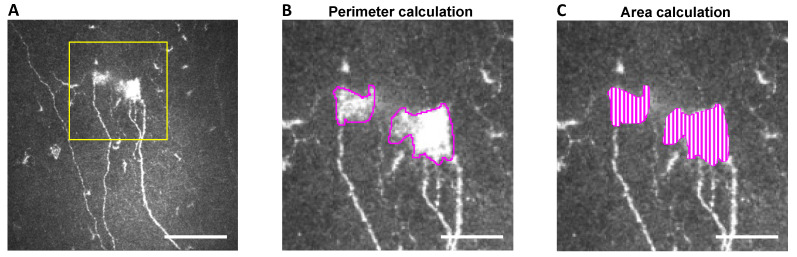
Microneuroma perimeter and area calculation methodology using IVCM images. Illustration of microneuroma analysis (yellow square): (**A**) The perimeter of microneuroma is delimited using ImageJ, (**B**) and the area is subsequently calculated (**C**) based on previous perimeter delimitation. Scale bar = 100 μm for (**A**) and 40 μm for (**B**,**C**).

Representative, high-quality images were chosen for patients of each study group, selecting only images with the subbasal plexus clearly in focus. To limit interoperator variability, a single investigator (A.G.M.) analyzed all the images using ImageJ (National Institutes of Health, Bethesda, MD) and assessed the following corneal ultrastructural parameters. Dendritiform cell density was calculated at the level of the Bowman layer to assess corneal inflammation, as previously described [29,33], using cell counter plugging from ImageJ (median (range): 15 (11–18) representative images/patient). The morphological study of the corneal nerves was analyzed by the following parameters: (1) nerve tortuosity classified in four grades according to an *ad hoc* tortuosity scale (Figure 2) (median (range): 77 (31–193) images/patient); (2) density of nerves (µm/mm^2^) was calculated using only images with clear uninterrupted visualization of the corneal nerves and less than 20% overlap between slices, to avoid any miscounts (median (range):11 (5–31) representative images/patient). These images were analyzed using the NeuronJ plugin in ImageJ; (3) number, area, and perimeter of microneuromas (Figure 3); microneuromas were defined as nerve abnormalities that present as irregularly shaped, hyperreflective, terminal enlargements of subbasal nerve endings, according to the relevant literature [27,31] (median (range): 77 (31–193) images/patient).

### 2.5. Statistical Analyses

Statistical analyses were performed using SPSS v.23 (SPSS Inc., Chicago, IL, USA). The normal distribution of the variables was tested independently in each study group using the Kolmogorov–Smirnov test. Parametric variables were compared with a *t*-test, non-parametric variables with Mann–Whitney U test, and nominal variables with χ^2^. Graphical representations were made using GraphPad Prism v.9 (GraphPad Software Inc., La Jolla, CA, USA). Data were expressed as mean ± SD.

## 3. Results

### 3.1. Clinical Variable Comparisons between Painful and Painless DED Patients

In the first part of the study, we investigated possible differences in signs and symptoms between patients suffering from MGD and AIDE both in painless patients (VAS = 0) and in patients with severe pain (VAS ≥ 8). Out of the 921 patients, 238 fit the initial inclusion criteria, with 122 cases of AIDE and 116 cases of primary MGD selected for patient clustering (Figure 1, Table 1). The mean age of patients was 56.8 ± 15.4 years, and 83% were female. A female predominance was observed in the AIDE group, compared with MGD both in the painless (VAS = 0; *p* < 0.05) and painful (VAS ≥ 8; *p*< 0.01) conditions. Demographic parameters are summarized in Table 1.

### 3.2. Higher Symptoms and Signs in Painful DED Patients Compared with Their Respective Painless Counterparts

Compared with their painless counterparts, painful DED (both MGD and AIDE) patients had significantly higher OSDI scores (*p* < 0.0001) and reported significantly more itching (*p* < 0.001), burning (*p* < 0.01 for MGD and *p* < 0.001 for AIDE) and foreign body sensations (*p* < 0.0001) (Table 2). Dryness sensation was significantly more often reported in painful AIDE, compared with painless AIDE (*p* < 0.0001) but not in painful MGD, compared with painless MGD (Table 2). In the MGD group, corneal damage (assessed by Oxford score) was significantly higher in painful MGD, compared with painless MGD (*p* < 0.01) (Table 3).

### 3.3. Higher Symptoms and Signs in Painful AIDE Patients Compared with Painful MGD

Patients suffering from painful AIDE presented significantly more foreign body sensations and dryness sensations, compared with painful MGD (*p* < 0.05) (Table 2). This was associated with significantly more corneal damage (higher Oxford scores; darker colors in Table 3) in painful AIDE patients, compared with painful MGD (*p* < 0.01). An important finding was that 71 % of the patients with painful MGD had low (0–1) Oxford score, while in patients with painful AIDE, the percentage was 42 % (Table 3). Regarding tear quality, painful MGD patients had significantly longer BUT and average NIKBUT, compared with painful AIDE patients (*p* < 0.01) (Table 3).

### 3.4. Higher Symptoms and Signs in Painless AIDE Compared with Painless MGD

Similarly, patients with painless AIDE had significantly higher OSDI scores (*p* < 0.05) and more foreign body or burning sensations than patients with painless MGD (*p* < 0.05) (Table 2). This was associated with significantly more corneal staining in painless AIDE patients, compared with painless MGD patients (*p* < 0.0001) (Table 3).

### 3.5. IVCM Image Analysis

In the second part of the study, DED patients were clustered into different groups (see Section 2.2.2. and Figure 1 for details)—namely, AIDE-related NCP (7 patients) and MGD-related NCP (11 patients), compared with painless AIDE (8 patients) and painless MGD (8 patients), respectively, and paired healthy controls (10 subjects). Dendritiform cell density and corneal nerve alterations (density, tortuosity, and microneuromas), detected using IVCM, were compared between the study groups.

### 3.6. DED-Related NCP Patients Have Significant IVCM-Identified Corneal Nerve Alterations Compared with Healthy Controls

**Figure 4:**
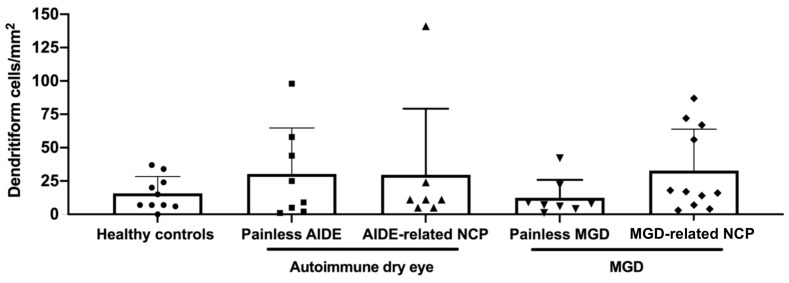
Dendritiform cell density (expressed as the number of cells per mm^2^) in the various study groups. Intergroup comparisons were made using Mann–Whitney U test, without any statistically significant differences between any of the study groups.

**Figure 5:**
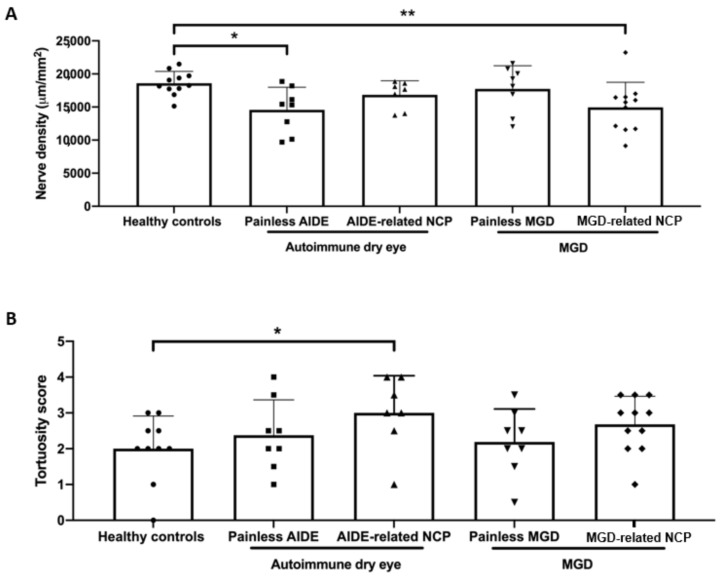
(**A**) Corneal nerve density (expressed as total nerve length in μm per mm^2^) in the various study groups. Intergroup comparisons were made using an unpaired *t*-test; (**B**) corneal nerve tortuosity (expressed as mean tortuosity score, as defined in Figure 2). Intergroup comparisons were made using Mann–Whitney U test. Statistical significance was illustrated as follows: * *p* < 0.05, ** *p* < 0.01.

**Figure 6:**
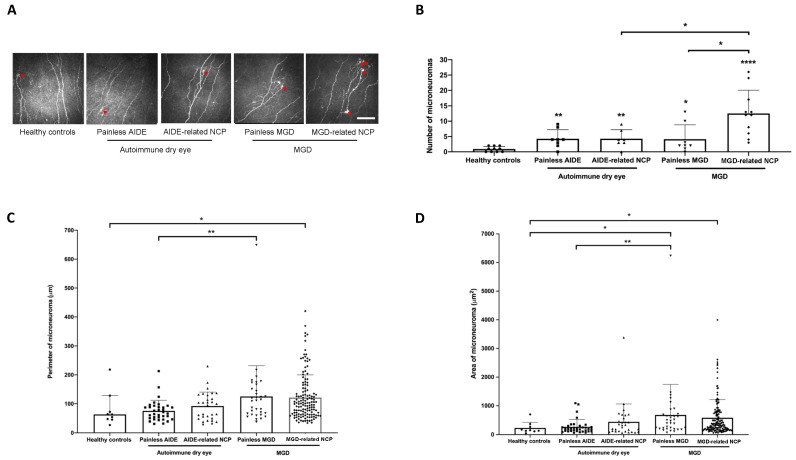
(**A**) In vivo confocal microscopy images evidencing microneuromas (red arrowheads) in both painful neuropathic conditions (MGD-related NCP and AIDE-related NCP), both painless DED control conditions (painless MGD and painless AIDE), and healthy volunteers. Scale bar = 100 μm; (**B**) microneuroma quantification (expressed as the number of microneuromas per patient in each study group); (**C**) microneuroma perimeter (expressed as the perimeter in μm for each microneuroma of each patient); (**D**) microneuroma area (expressed as the area in μm^2^ for each microneuroma of each patient). Intergroup comparisons were made using Mann–Whitney U test. Statistical significance was illustrated as follows: * *p* < 0.05, ** *p* < 0.01, **** *p* < 0.0001 versus control group; except when a bar line is used.

No significant differences could be observed between the various study groups regarding dendritiform cell density (Figure 4), suggesting limited corneal inflammation in the present experimental setting. MGD-related NCP patients had significantly lower nerve density (*p* < 0.01) (Figure 5A), a higher number of microneuromas per patient (*p* < 0.0001) (Figure 6A,B), higher microneuroma perimeter (*p* < 0.05) (Figure 6C) and area (*p* < 0.05) (Figure 6D), compared to healthy controls. Similarly, AIDE-related NCP patients had significantly higher corneal nerve tortuosity (*p* < 0.05) (Figure 5B) and number of microneuromas per patient, compared to healthy controls (*p* < 0.01) (Figure 6B). Collectively, these results suggest that the patients defined in this study as having DED-related NCP (“pain with no stain”) did indeed show neuropathic corneal nerve alterations. This conclusion is further supported by the limited inflammation indicated by the low dendritiform cell density in all experimental conditions (Figure 4), suggesting limited (if any) involvement of nociceptive inflammatory mechanisms in the corneal nerve alterations identified in IVCM.

### 3.7. MGD-Related NCP Patients Had Higher Microneuromas Compared with Painless MGD and AIDE-Related NCP Patients

Interestingly, MGD-related NCP patients had a significantly higher number of microneuromas per patient compared to painless MGD patients (*p* < 0.05) and AIDE-related NCP patients (*p* < 0.05) (Figure 6B), suggesting that MGD could induce substantial corneal nerve alterations and possibly neuropathic pain development, more so than AIDE diseases.

### 3.8. Higher Microneuroma Found in Painless DED Patients Compared with Healthy Controls

Even in the absence of a neuropathic pain phenotype, microneuromas were observed in painless DED patients. Indeed, painless MGD patients had a significantly higher number of microneuromas per patient (*p* < 0.05) (Figure 6B) and higher microneuroma area (*p* < 0.05) (Figure 6D), compared with healthy patients, and similarly, painless AIDE patients had significantly lower nerve density (*p* < 0.05) (Figure 5A) and a significantly higher number of microneuromas per patient (*p* < 0.01) (Figure 6B), compared to healthy controls.

### 3.9. Structural Differences in Microneuromas between Painless MGD Patients and AIDE Patients

Finally, microneuroma perimeter (Figure 6C) and area (Figure 6D) were both significantly higher in painless MGD patients than painless AIDE (*p* < 0.01), suggesting that such IVCM parameters (area and perimeter) could help differentiate corneal nerve alterations in these two subsets of DED patients.

## 4. Discussion

DED, frequently observed in ophthalmological practice, affects between 5 and 50% of the adult population and is gaining recognition as a public health issue, given its prevalence, morbidity, and associated financial burden [1,2,3,4].

DED is a heterogeneous clinical condition with growing evidence of underlying neurosensory abnormalities [1]. Furthermore, increasing preclinical and clinical lines of evidence suggest that DED could lead to NCP, a still ill-defined, high burden, painful ocular condition with few efficient treatment options [14,31]. Understanding NCP is thus of paramount clinical importance.

Significant heterogeneity could exist between DED subsets, such as AIDE and primary MGD, in terms of ocular surface symptomatology, semiology, and corneal nerve alterations, as evidenced in this study. Interestingly, the most important ultrastructural corneal nerve changes were found in the MGD-related NCP group—namely, decreased nerve density, higher number of microneuromas and increased microneuroma perimeter and area, even compared with the rest of DED groups. These observations suggest, for the first time, that primary MGD could be a previously unsuspected purveyor of NCP. Therefore, patients reporting severe ocular pain with mild-to-no corneal damage (aptly referred to as “pain with no stain”) in cases of primary MGD should be considered as possibly suffering from NCP and managed accordingly [2,14].

Compared with the previous literature in the field, this study is the first to analyze the clinical profiles of these painful DED patients. We found that overall, painless or painful AIDE groups were more severe than corresponding MGD groups; the painful groups (AIDE and MGD) had higher symptoms and signs scores than the painless ones. Painful neuropathic conditions are notoriously difficult to manage, and NCP is no exception. Such difficulties in management stem from both complex diagnosis (in the absence of validated diagnostic criteria) and partially effective treatments with unpredictable results [2,15,34]. Regarding the former, several authors have investigated possible biomarkers of NCP, suggesting for instance that the sole presence of IVCM-identified corneal nerve microneuromas could be indicative of neuropathic pain [31]. Results from this study tend to disprove such a claim, as microneuromas were observed in painless DED patients and even in healthy subjects (Figure 6B), as recently reported [35].

Although microneuromas do bear witness of corneal nerve abnormalities [27,36], the latter seem insufficient to produce neuropathic pain. Therefore, from a diagnostic standpoint, the mere presence of microneuromas seems insufficient to suggest corneal neuropathic alterations. Additional IVCM parameters, such as microneuroma perimeter and area, could thus be necessary, which in this experimental setting were both significantly higher in MGD-related NCP than in healthy subjects (Figure 6C,D).

There is growing evidence in the literature suggesting high phenotypic diversity, evidenced as different sensory profiles in painful neuropathic conditions with the same underlying etiology that could account for the often-observed partial treatment response in the clinical setting [32]. Therefore, phenotyping neuropathic pain could help in providing tailored treatment options with higher effectiveness and lower untoward effects [32,37,38]. In the present context of DED-related NCP, IVCM identified significantly diverse corneal nerve alterations, different between the painful neuropathic and painless conditions but also between the two DED subsets, suggestive of such aforementioned neuropathic phenotypic diversity. Based on data from the relevant literature [2,19,31,39,40] and from the results of this study, DED-related NCP patients could be stratified based on IVCM-identified corneal nerve alterations, as illustrated in Table 4.

Further studies will be needed to determine whether this stratification provides new insights into the management of NCP.

The precise causal relationship between DED symptomatology/semiology and IVCM-identified corneal nerve abnormalities also warrants future studies to elucidate this “chicken-or-egg” conundrum. Indeed, DED has been shown to lead to NCP (with associated corneal nerve alterations), and conversely, NCP often presents as a “dry eye” sensation [1,9].

Several limitations in the present study warrant discussion. First, the retrospective nature of the study and small sample size are obvious limitations, at least partially, mitigated by nested paired group analysis and proper patient clustering. Second, DED subsets were divided arbitrarily between primary MGD and all AIDE patients. This was voluntary to provide cohorts of comparable sizes separated by major underlying pathophysiological mechanisms (i.e., ADDE vs. EDE) even though overlap is most probable in several conditions (such as in AIDE patients who develop secondary MGD). Future multicentric studies are warranted to provide better clustering between DED subsets. Finally, there was a limited number of patients in each final study group (after appropriate clustering), which could hamper the proper interpretation of IVCM results. Nevertheless, considering the rigorous IVCM image analysis methodology and the high number of images analyzed per patient (to our knowledge, never included in earlier studies), we believe our results to be sound and thus worthy of consideration.

## 5. Conclusions

Patients suffering from primary MGD-related NCP could present a different neuropathic phenotype, compared to the one observed in AIDE-related NCP patients, as proven herein using IVCM. Specifically, primary MGD-related NCP was associated with significantly more ultrastructural corneal nerve alterations than AIDE-related NCP or healthy controls. From a diagnostic standpoint, although IVCM can be useful to evidence NCP-related corneal nerve changes in DED patients, the diagnosis of DED-related NCP will require an association of several IVCM-based criteria without relying solely on the presence of microneuromas.

## Figures and Tables

**Table 1:** Demographic parameters. Intergroup comparisons were made with *t*-test (age) and χ^2^ test (gender).

			Autoimmune Dry Eye (AIDE)		Meibomian Gland Dysfunction (MGD)	
		All Patients in the Study	Painless	Painful	Painless	Painful
VAS		-	0	8–10	0	8–10
Number of patients (%)		238 (100%)	63 (27%)	59 (25%)	63 (27%)	53 (22%)
Age (mean ± SD)		56.8 ± 15.4	58.1 ± 13.8	57.0 ± 15.0	55.4 ± 16.0	56.8 ± 16.9
Gender (n, %)	Male	40 (17%)	6 (10%)	4 (7%)	16 (25%)	14 (26%)
	Female	198 (83%)	57 (91%) †	55 (93%) ‡‡	47 (75%)	39 (74%)

† Significant difference between painless AIDE vs. painless MGD, ‡ Significant difference between painful AIDE vs. painful MGD. Statistical significance is illustrated based on number of signs as follows: one sign = *p* < 0.05, two signs = *p* < 0.01.

**Table 2:** Ocular surface symptomatology. Intergroup and intragroup comparisons were made with Mann–Whitney U test.

			Autoimmune Dry Eye (AIDE)		Meibomian Gland Dysfunction (MGD)	
		All Patients in the Study	Painless	Painful	Painless	Painful
OSDI score (mean ± SD)		62.5 ± 24.8	57.5 ± 25.7 †	76.8 ± 15.1 ####	46.6 ± 24.1	70.8 ± 21.0 ●●●●
Itching (n, %)	Never	115 (49%)	38 (62%)	20 (34%)	35 (56%)	22 (42%)
	Rarely	44 (19%)	10 (16%)	8 (14%)	17 (27%)	9 (17%)
	Sometimes	51 (22%)	10 (16%)	21 (36%)	8 (13%)	12 (23%)
	Always	26 (11%)	3 (5%)	10 (17%) ###	3 (5%)	10 (19%) ●●●
Burning (n, %)	Never	66 (28%)	22 (35%)	4 (7%)	35 (57%)	5 (9%)
	Rarely	39 (16%)	18 (29%)	6 (10%)	9 (14%)	6 (11%)
	Sometimes	86 (36%)	18 (29%)	31 (52%)	15 (24%)	22 (41%)
	Always	46 (19%)	5 (8%) †	18(30%) ####	3(5%)	20 (38%) ●●
Foreign body sensation (n, %)	Never	73 (31%)	21 (33%)	5 (8%)	32 (52%)	15 (28%)
	Rarely	38 (16%)	15 (24%)	5 (8%)	11 (18%)	7 (13%)
	Sometimes	84 (35%)	17 (27%)	33 (56%)	13 (21%)	21 (40%)
	Always	42 (18%)	10 (16%) †	16 (27%) ####, ‡	6 (10%)	10 (19%) ●●●●
Dryness sensation (n, %)	Never	94 (39%)	28 (44%)	11 (19%)	33 (52%)	22 (42%)
	Rarely	32 (13%)	14 (22%)	6 (10%)	6 (10%)	6 (11%)
	Sometimes	71 (30%)	15 (24%)	23 (39%)	19 (30%)	14 (26%)
	Always	41 (17%)	6 (10%)	19 (32%) ####, ‡	5 (8%)	11 (21%)

# significant difference between painless AIDE vs. painful AIDE; ● significant difference between painless MGD vs. painful MGD; † significant difference between painless AIDE vs. painless MGD; ‡ significant difference between painful AIDE vs. painful MGD. Statistical significance is illustrated based on number of signs as follows: one sign = *p* < 0.05, two signs = *p* < 0.01, three signs = *p* < 0.001, four signs = *p* < 0.0001.

**Table 3:** Ocular surface semiology. Intergroup comparisons were made with Mann–Whitney U test (Oxford score, BUT, NIKBUT (first), Schirmer, tear meniscus, osmolarity) and *t*-test (NIKBUT (average)).

		Autoimmune Dry Eye (AIDE)		Meibomian Gland Dysfunction (MGD)	
	All Patients in the Study	Painless	Painful	Painless	Painful
Oxford score 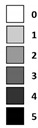	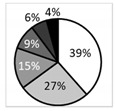	†††† 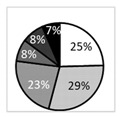	‡‡ 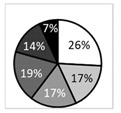	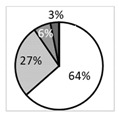	●● 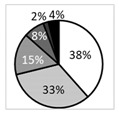
BUT (s, mean ± SD)	4.94 ± 0.18	4.82 ± 0.35	4.19 ± 0.31	5.70 ± 0.36	5.00 ± 0.39 ‡
NIKBUT (first, mean ± SD)	5.06 ± 5.35	4.08 ± 4.77	3.55 ± 3.65	5.83 ± 5.14	6.66 ± 7.45
NIKBUT (average, mean ± SD)	7.78 ± 6.90	6.8 ± 6.52	5.10 ± 5.41	9.24 ± 6.87	9.27 ± 8.18 ‡
Schirmer (mm/5 min, mean ± SD)	10.9 ± 10.0	10.1 ± 10.1	8.8 ± 9.1	12.8 ± 10.6	12.0 ± 9.8
Tear meniscus (mm, mean ± SD)	0.209 ± 0.188	0.172 ± 0.198	0.224 ± 0.193	0.235 ± 0.200	0.196 ± 0.141
Osmolarity (mOsmol/L, mean ± SD)	309 ± 17	307 ± 15	311 ± 19	306 ± 16	310 ± 19

● significant difference between painless MGD vs. painful MGD; † significant difference between painless AIDE vs. painless MGD; ‡ significant difference between painful AIDE vs. painful MGD. Statistical significance is illustrated based on number of signs as follows: one sign = *p* < 0.05, two signs = *p* < 0.01, four signs = *p* < 0.0001.

**Table 4:** Comparison of in vivo confocal microscopy-assessed corneal ultrastructural characteristics associated with NCP.

		IVCM-Assessed Corneal Ultrastructural Characteristics (vs. Healthy Controls)					
		↘ Nerve Density	↗ Nerve Tortortuosity	↗ Number of Dendritic Cells	↗ Number of MN	↗ Perimeter of MN	↗ Area of MN
MGD	MGD-NCP	**✓**	ns	ns	**✓**	**✓**	**✓**
	Painless	ns	ns	ns	**✓**	ns	**✓**
AIDE	AIDE-NCP	ns	**✓**	ns	**✓**	ns	ns
	Painless	**✓**	ns	ns	**✓**	ns	ns

↘ decrease; ↗ increase; ns: non-significant difference (vs. healthy controls); **✓** significant difference (vs. healthy controls).

## Data Availability

The datasets used and/or analyzed during the current study are available from the corresponding author on reasonable request.
